# Leupaxin stimulates adhesion and migration of prostate cancer cells through modulation of the phosphorylation status of the actin-binding protein caldesmon

**DOI:** 10.18632/oncotarget.3792

**Published:** 2015-04-12

**Authors:** Sascha Dierks, Sandra von Hardenberg, Thomas Schmidt, Felix Bremmer, Peter Burfeind, Silke Kaulfuß

**Affiliations:** ^1^ Institute of Human Genetics, University Medical Center Göttingen, Germany; ^2^ Center of Pharmacology and Toxicology, Hannover Medical School, Germany; ^3^ Department of Anatomy, University of Witten/Herdecke, Witten, Germany; ^4^ Institute of Pathology, University Medical Center Göttingen, Germany

**Keywords:** prostate cancer, leupaxin, caldesmon, migration

## Abstract

The focal adhesion protein leupaxin (LPXN) is overexpressed in a subset of prostate cancers (PCa) and is involved in the progression of PCa. In the present study, we analyzed the LPXN-mediated adhesive and cytoskeletal changes during PCa progression. We identified an interaction between the actin-binding protein caldesmon (CaD) and LPXN and this interaction is increased during PCa cell migration. Furthermore, knockdown of LPXN did not affect CaD expression but reduced CaD phosphorylation. This is known to destabilize the affinity of CaD to F-actin, leading to dynamic cell structures that enable cell motility. Thus, downregulation of CaD increased migration and invasion of PCa cells. To identify the kinase responsible for the LPXN-mediated phosphorylation of CaD, we used data from an antibody array, which showed decreased expression of TGF-beta-activated kinase 1 (TAK1) after LPXN knockdown in PC-3 PCa cells. Subsequent analyses of the downstream kinases revealed the extracellular signal-regulated kinase (ERK) as an interaction partner of LPXN that facilitates CaD phosphorylation during LPXN-mediated PCa cell migration. In conclusion, we demonstrate that LPXN directly influences cytoskeletal dynamics via interaction with the actin-binding protein CaD and regulates CaD phosphorylation by recruiting ERK to highly dynamic structures within PCa cells.

## INTRODUCTION

Prostate cancer is the most common cancer type and the third most common cause of cancer-related death among men in developed countries [1]. Many prostate tumors are locally defined and slowly growing and can be successfully treated by surgery, radiation or hormone therapy. Because these therapies are often accompanied with urinary or sexual dysfunction, active surveillance is the preferred alternative therapy for patients who suffer from locally defined prostate cancer. However, some of these prostate tumors will progress, producing the metastatic stage, which is associated with a poor prognosis and often results in cancer-related death.

Cancer cell adhesion has been known to be a critical feature of this metastatic spread, and understanding the underlying mechanisms of metastasis in PCa will result in the identification of biomarkers for the advanced stages of PCa. Reduction of focal adhesions and their high turnover is required for cell motility, cell migration and cell invasion. Focal adhesions are important cellular structures that build a structural link between the extracellular matrix (ECM) and the actin cytoskeleton. Their components take part in cell signaling downstream of integrin activation induced by ECM proteins such as fibronectin and collagen [2].

One of those focal adhesion proteins is leupaxin (LPXN), which belongs to the paxillin protein family. Characterized by their subcellular localization at focal adhesion sites and their specific domain structure composed of LIM domains and LD motifs, paxillin family proteins are mainly known for protein-protein interactions [3]. Originally, LPXN was found in cells of hematopoietic origin [4]. Later, LPXN was found to be an important adapter protein in osteoclasts during the formation of adhesion zones. The disassembly of LPXN from the podosomal signaling complex inhibits the migratory and resorptive ability of osteoclasts [5]. Furthermore, LPXN is also enriched in vascular smooth muscle cells, where it has been shown to regulate migratory and contractile properties during vasculogenesis in a FAK/SRC-dependent manner [6].

Recently, we found that LPXN was overexpressed in approximately 20% of prostate cancer patients and that LPXN expression correlated with the differentiation stage of these tumors [7]. Downregulation of LPXN expression in the PCa cell lines resulted in reduced migration and invasion. Using the transgenic LPXN/TRAMP (transgenic adenocarcinoma of mouse prostate) mouse model with prostate-specific LPXN expression, we showed that LPXN enhances PCa progression [8]. In addition, LPXN was shown to form a signaling complex with PYK2, FAK, c-SRC, and PTP-PEST [9]. These interactions regulate the phosphorylation status and localization of focal adhesion-associated proteins such as LPXN to ensure proper focal adhesion formation and migration of PCa cells. However, none of these interaction partners has been shown to directly affect the stability of the actin cytoskeleton.

One protein that is essential for this process and during the regulation of smooth muscle and non-muscle contraction is the protein caldesmon 1 (CaD, *CALD1*). CaD is known to regulate the actin-myosin interaction and thereby actin cytoskeletal stabilization in a Ca^2+^/Calmodulin and phosphorylation-dependent manner [10, 11]. The *CALD1* gene encodes five different CALD1 transcripts, resulting in two major isoforms: a high-molecular-mass isoform (h-CaD) that is expressed in smooth muscle cells and a low-molecular-mass isoform (l-CaD) expressed in non-muscle cells. The regulation of CaD is important for proper cell function because decreased expression of l-CaD has been found in many cancer cell types [12-15].

In the present study, we identify the actin-binding protein CaD as a new interaction partner of LPXN, thereby linking LPXN directly to the actin cytoskeleton for the first time. Furthermore, we provide a novel mechanism for the regulation of the actin cytoskeleton during migration: LPXN-mediated phosphorylation of CaD by the extracellular-signal regulated kinase 1/2 (ERK).

## RESULTS

### Reduced adhesion and cell size of PCa cells after LPXN knockdown

**Figure 1:**
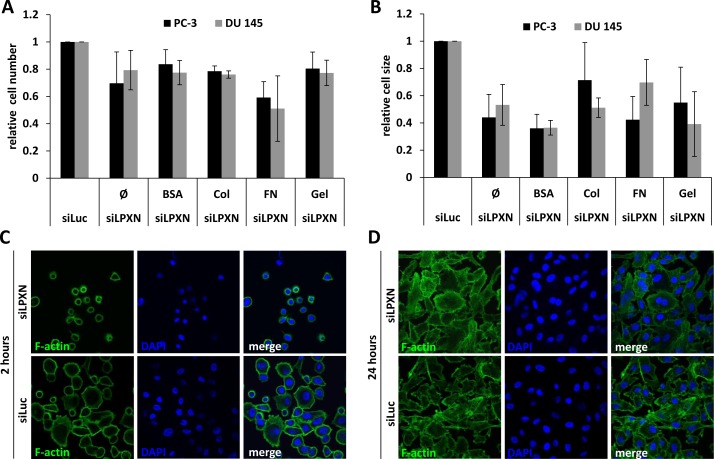
LPXN knockdown decreases adhesion and cell size To analyze adhesion, PC-3 and DU 145 cells transfected with siRNA against LPXN (siLPXN) or luciferase (siLuc = control) were plated on glass culture slides that were either uncoated (Ø) or coated with bovine serum albumin (BSA), collagen (Col), fibronectin (FN) or gelatin (Gel). Cells were fixed 2 hours after plating; the cytoskeleton was visualized using FITC-conjugated phalloidin (green), and nuclei were stained with DAPI (blue). Cell number (**A**) and cell size (**B**) were determined by confocal microscopy. After 2 hours (**C**) of adhesion, siLPXN-transfected cells showed a reduced surface area compared to control-transfected cells, whereas 24 hours (**D**) later, they were not distinguishable from each other.

To investigate the influence of LPXN expression on the adhesive characteristics of PCa cells, we performed a cell adhesion assay. After downregulation of LPXN expression in PC-3 and DU 145 cells using a specific siRNA, cells were plated on glass slides coated with fibronectin (FN), rat tail collagen (Col), bovine serum albumin (BSA) or gelatin (Gel). Adhered cells were fixed after 2 hours of incubation, and the cytoskeleton was visualized using FITC-conjugated phalloidin. Cell numbers and cell size were analyzed using confocal fluorescence microscopy. We observed that cells with LPXN knockdown showed reduced adhesion on all substrates in comparison to control cells (Figure 1A). The strongest effect of LPXN knockdown was observed for adhesion on FN-coated slides. In addition, the highest difference in cell size between LPXN knockdown and control transfected (siLuc) cells was observed on FN-coated and BSA-coated slides (Figure 1B). Thus, loss of LPXN expression seems to reduce the capability to adhere to the ECM in PCa cells.

As summarized in Figure 1C, PC-3 cells showed a significantly reduced surface area after LPXN knockdown compared with control transfected cells. After 2 hours, control cells were already spread on the substratum and had a strong contact to the fibronectin matrix, whereas cells with LPXN knockdown remained rounded and showed no cell protrusions. As a control and to study the effect of LPXN knockdown on long-term adhesion, cells transfected with siLPXN or siLuc (control) were allowed to adhere for 24 hours. During this time course, both cell populations could completely adhere to the substratum and showed no difference in their morphology (Figure 1D), pointing to a function of LPXN in early adhesion dynamics.

### LPXN interacts with the actin-binding protein CaD

**Figure 2:**
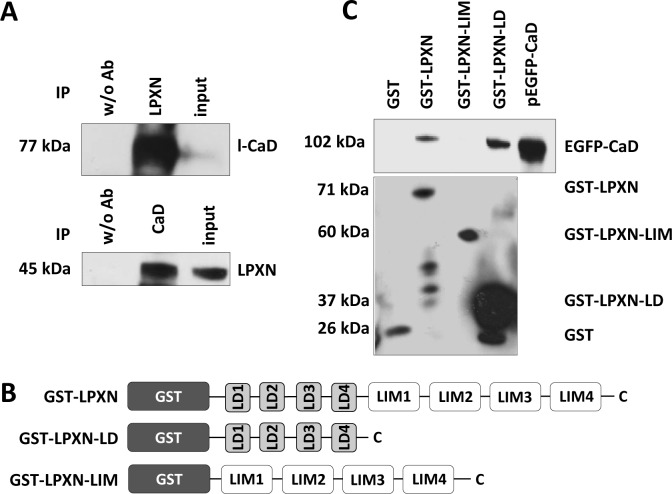
LPXN interacts with CaD (**A**) Co-immunoprecipitation of LPXN and l-CaD. Immunoprecipitation using a LPXN-specific antibody showed a specific band at 77 kDa for l-CaD in the corresponding western blot. Reciprocal immunoprecipitation using a CaD-specific antibody revealed a specific band at 45 kDa in the immunoblot using a LPXN-specific antibody. Control lysates did not show precipitation. Input served as a positive control. (**B**) Schematic overview of the constructs used in the GST pulldown assay. GST was always fused to the N-terminal site of the LPXN fragments. GST-LPXN contained the full length LPXN, GST-LPXN-LD fused the four LD-motifs to the GST protein and GST-LPXN-LIM carried only the four LIM-domains. (**C**) PC-3 cells were transfected with EGFP-CaD, and protein lysates were subjected to a GST pulldown assay with the depicted GST-LPXN fusion proteins. Subsequently, western blot analysis with a CaD-specific antibody was performed to show the interaction of LPXN with CaD. Lysate of PC-3 cells transfected with pEGFP-CaD served as a positive control. GST antibody showed the presence of the corresponding fusion proteins: GST (26 kDa), GST-LPXN (71 kDa), GST-LPXN-LIM (60 kDa), and GST-LPXN-LD (37 kDa). Interaction of LPXN with CaD is dependent on the LD motifs of LPXN. No interaction was observed using LPXN-LIM.

To identify proteins that could facilitate the cytoskeletal changes mediated by LPXN, we performed a yeast two-hybrid screen using a human prostate cDNA library with full-length LPXN as bait. This resulted in two different clones encoding the human actin-binding protein caldesmon (CaD, *CALD1*). Subsequently, we verified the specificity of this interaction by a direct yeast two-hybrid experiment with full-length constructs of LPXN and CaD (Supplemental Figure 1). The presence of the corresponding pGBKT-7-LPXN and pGADT-7-CALD1 plasmids was confirmed by PCR analyses (Supplemental Figure 1). Furthermore, the interaction between CaD and LPXN was validated by a co-immunoprecipitation assay (Figure 2A). The immunoblot showed a specific band at 45 kDa, meaning that LPXN was precipitated from the lysate of PC-3 cells. Control cells did not show precipitation of LPXN. In addition, reciprocal co-immunoprecipitation with a LPXN-specific antibody confirmed the interaction of LPXN with CaD (Figure 2A).

Next, we conducted a series of glutathione S-transferase (GST) pulldown assays to identify the LPXN protein domains responsible for this interaction. Therefore, PC-3 cells were transfected with EGFP-CALD1. Several LPXN fusion proteins were used to pull down EGFP-CALD1 from isolated PC-3 protein lysates: GST-LPXN, containing full-length LPXN, GST-LPXN-LD, harboring only the four LD motifs of LPXN (aa 32-176) and GST-LPXN-LIM, harboring only the four LIM domains of LPXN (aa 173-417; Figure 2B). A specific signal at 102 kDa for EGFP-CaD was detected using GST-LPXN and GST-LPXN-LD, indicating a direct interaction between CaD and LPXN, specifically with the LD motifs of LPXN (Figure 2C).

### Expression and subcellular localization of CaD in PCa cell lines

**Figure 3:**
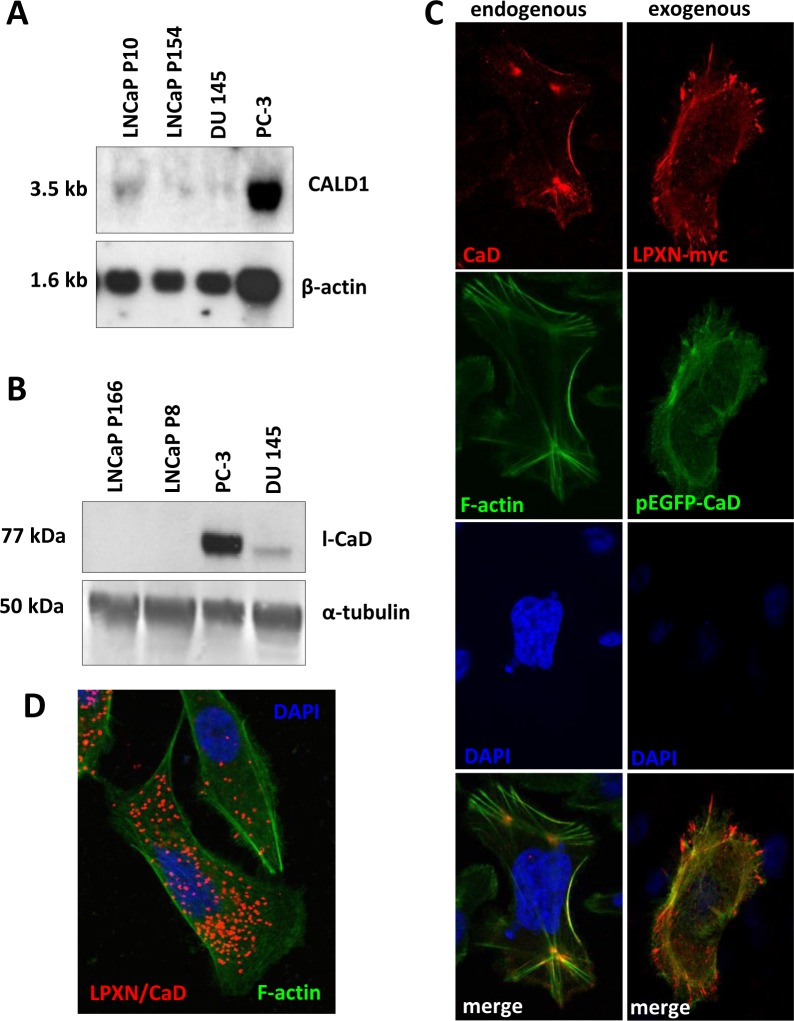
Expression and localization of CaD in PCa cell lines (**A**) Northern blot analysis of PC-3, DU 145 and LNCaP high- and low-passage cells shows that the highest CaD expression occurred in PC-3. β-actin served as a loading control. (**B**) Western blot analysis of PC-3, DU 145 and LNCaP cells shows the highest l-CaD expression in PC-3 and very weak levels of l-CaD in DU 145 cells. (**C**) Immunofluorescence staining of endogenous CaD showing co-localization with the actin cytoskeleton. Transfection of PC-3 cells with LPXN-myc and EGFP-CaD fusion constructs shows co-localization of CaD and LPXN. (**D**) Proximity ligation assay using specific CaD and LPXN antibodies showing that these interactions are localized to the sub-membranous compartment, whereas no interactions were present at the protrusion zone of migrating cells or at stabilized actin structures.

The ability of CaD to bind and stabilize actin and the interaction with LPXN would suggest CaD as a putative modulator of actin-cytoskeletal remodeling in PCa cell lines. To analyze the expression of CaD in the well-established PCa cell lines PC-3, DU 145 and LNCaP, we performed northern blot and western blot analyses. At both the RNA and protein levels, we detected the strongest expression of CaD in the androgen-independent and invasive PCa cell line PC-3 (Figure 3A and 3B). Weak expression of CaD was found in androgen-independent and invasive DU 145 cells; in the non-invasive and androgen-dependent low-passage LNCaP cells and in the non-invasive and androgen-independent high-passage LNCaP cells, CaD expression was only observed on the RNA level. Interestingly, in previous studies, we demonstrated the same expression pattern of LPXN in these cell lines at the protein level [7].

To investigate the subcellular localization of CaD, PC-3 cells were plated on glass slides and fixed after 24 hours, followed by immunofluorescent detection using a CaD-specific antibody. As shown in Figure 3C, CaD was mainly localized at the F-actin filaments.

To examine the subcellular co-localization of CaD and LPXN by immunocytochemistry, PC-3 cells were co-transfected with pEGFP-CALD1 and pCMV-LPXN-cmyc fusion constructs. Detection of the LPXN-cmyc fusion protein using a cmyc-specific antibody revealed co-localization of LPXN (red) and CaD (green) at focal adhesion sites and the F-actin filaments (Figure 3C).

Having shown a clear interaction between LPXN and the actin-binding protein CaD, we investigated the sites of LPXN-CaD interaction through an *in situ* proximity ligation assay (PLA) on PC-3 cells using specific LPXN and CaD antibodies, respectively. Interaction of the two proteins is indicated by the red dots (Figure 3D). Confocal fluorescence microscopic analysis of the PLA revealed that LPXN-CaD interaction was mainly localized to the sub-membranous compartments, whereas no interaction was detected at the protrusion zone of migrating cells or at stabilized actin structures and podosomes (Figure 3D). We observed little interaction of LPXN and CaD in non-migrating or quiescent PCa cells, indicating the involvement of LPXN in CaD regulation.

### Downregulation of CaD expression in PCa cells stimulates cell migration

**Figure 4:**
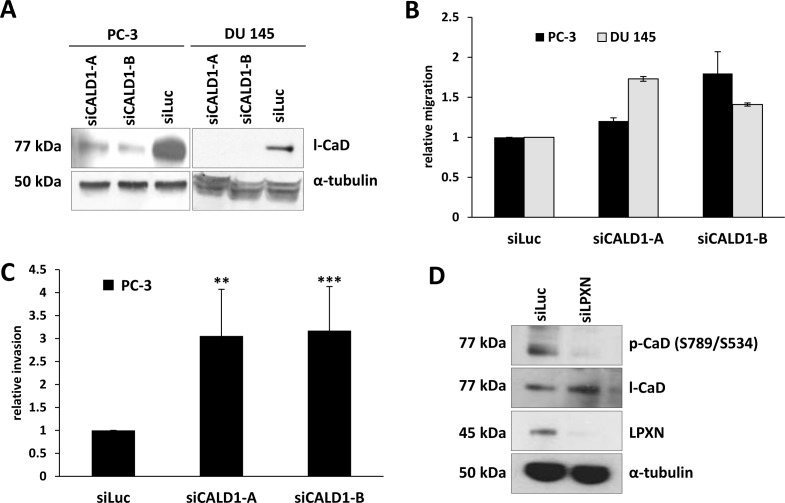
Knockdown of CaD increases cell migration and invasion (**A**) PC-3 and DU 145 cells were transfected with two different siRNAs specific for CaD (siCALD1-A and siCALD1-B) and one as a control for the luciferase gene (siLuc). Knockdown of l-CaD was confirmed by western blot using a CaD-specific antibody. Equal protein loading was confirmed using α-tubulin. (**B**, **C**) PC-3 and DU 145 cells were transfected with CaD-specific siRNAs and incubated for 48 hours, followed by migration and invasion assays. Downregulation of CaD caused a significant increase in the migration of PC-3 and DU 145 cells as well as enhanced invasion of PC-3 cells. (**D**) PC-3 cells were transfected with LPXN-specific siRNA for 48 hours, serum-starved overnight and stimulated with 10% FCS for 4 hours. Protein lysates were subjected to western blot analysis to detect phosphorylated CaD. Knockdown of LPXN significantly reduced the phosphorylation status of l-CaD at S534 as shown by detection of a specific band at 77 kDa using a phosphorylation-specific antibody. Total l-CaD levels were not affected by LPXN knockdown. α-tubulin served as a loading control.

The low molecular mass CaD isoform (l-CaD) has been shown to play an important role in the formation and regulation of the actin- cytoskeletal network. To assess the function and relevance of CaD in PCa cells, CaD expression was downregulated in PC-3 and DU 145 cells using two different CALD1-specific siRNAs (siCALD1-A and siCALD1-B). qRT-PCR and western blot analyses showed a strong downregulation of CaD after siRNA treatment in comparison to control transfected cells (siLuc) (Figure 4A). A transwell migration assay analysis indicated that knockdown of CaD expression in the PC-3 and DU 145 cell lines results in a 2-fold increase in migratory capability (Figure 4B). Further, the invasion of PC-3 cells was increased 3-fold after knockdown of CaD (Figure 4C).

From the literature, CaD is also known to regulate the cell cycle by promoting M-phase entry [16]. To address this possibility, we analyzed the influence of CaD expression on cell proliferation in our PCa cell lines. Therefore, a proliferation assay was performed on PC-3 and DU 145 cells after CaD knockdown. The number of live cells was monitored over a time course of 72 hours. Downregulation of CaD expression in PC-3 and DU 145 cells was not significantly associated with any changes in cell proliferation (Supplemental Figure 2).

### LPXN knockdown has no influence on CaD expression but causes a reduction in phosphorylated CaD protein levels

Because it is known that LPXN can translocate to the nucleus and activate transcription factors such as the androgen receptor [7] and the serum response factor [6], we asked whether LPXN influences the expression of CaD. Therefore, LPXN was downregulated in PC-3 and DU 145 cells by RNAi. After 72 hours, cell lysates were isolated and immunoblotted with a CaD-specific antibody. As shown in Figure 4D, LPXN expression has no influence on CaD expression levels.

We further analyzed protein lysates that were isolated from LPXN overexpressing primary cells [8]. These cells were isolated from tumors of the prostate of double transgenic LPXN/TRAMP (Transgenic Adenocarcinoma of Mouse Prostate) mice. The isolated proteins, which were named according to the respective mice (83A and 72A), were immunoblotted with a CaD-specific antibody. As a control, we used proteins isolated from cultivated PCa primary cells of single transgenic TRAMP mice (27F and 45F), which do not show any overexpression of LPXN. Western blot analysis demonstrated that primary cells also show no LPXN-mediated regulation of CaD expression (Supplemental Figure 3). Therefore, neither downregulation nor overexpression of LPXN affected CaD expression levels.

The affinity of CaD for actin is regulated by phosphorylation and by binding of Ca^2+^-calmodulin [10, 17, 18]. Thus, phosphorylation status rather than expression might be important for CaD function in PCa cells. To address this possibility, we next examined the effect of LPXN knockdown on the phosphorylation status of CaD. At 48 hours after siLPXN transfection, PC-3 cells were serum-starved for 24 hours and then stimulated with serum for 4 hours. Cell lysate was immunoblotted with an anti-pS789 (S534)-specific antibody of CaD. This analysis indicated that LPXN knockdown does indeed lead to decreased levels of phospho-CaD compared to control (siLuc) transfected cells, whereas total CaD levels remained equal (Figure 4D). Thus, the LPXN interaction with CaD might regulate actin-cytoskeletal remodeling during prostate cancer progression.

### Increased interaction of LPXN and CaD during cell migration

**Figure 5:**
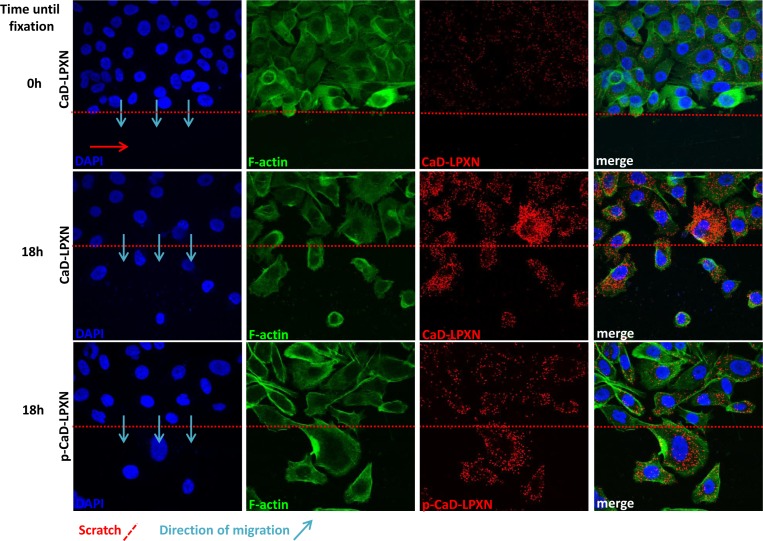
Interaction of CaD and LPXN during PCa cell migration Analysis of CaD-LPXN interactions during migration was shown by a combination of a scratch assay and a proximity ligation assay on PC-3 cells using specific CaD or pCaD-S534 and LPXN antibodies. Cells were grown until confluence, and a scratch was applied in the confluent cell layer. Confluent cells (upper row) did not show high numbers of interactions, whereas cells that migrated into the scratch showed increased interactions of CaD and LPXN (middle row) as well as pCaD and LPXN (lower row). Blue arrows indicate the direction of migration. The scratch is indicated by a red dotted line. Nuclei and F-actin were stained using DAPI and FITC-conjugated phalloidin, respectively.

We next investigated the biological relevance of the LPXN-CaD interaction during migration of PCa cells. Scratches were applied to confluent PC-3 cell layers. Cells were fixed immediately after scratching and after 18 hours of migration, and proximity ligation assays (PLA) were performed using LPXN- and CaD-specific antibodies. As summarized in Figure 5, we observed little LPXN-CaD interaction in confluent and non-migrating cells (Figure 5, upper panel), whereas strong interaction was observed in cells that were fixed during migration into the scratch (Figure 5, middle panel).

To study LPXN interaction with phosphorylated (pS534) CaD, a pS534-specific antibody was used in the PLA. Fluorescent microscopy also revealed increased interaction of phosphorylated CaD with LPXN in migrating cells (Figure 5, lower panel).

### LPXN influences the JNK and ERK pathways but not p38 MAPK signaling

As a multifunctional adapter protein that lacks kinase activity, LPXN may be able to interact with and recruit kinases that are responsible for CaD phosphorylation. There are several kinases known to phosphorylate CaD at specific sites and thereby regulate the actin binding of CaD. Among them are the extracellular signal-regulated kinase (ERK) and p38 MAPK [19, 20].

**Figure 6:**
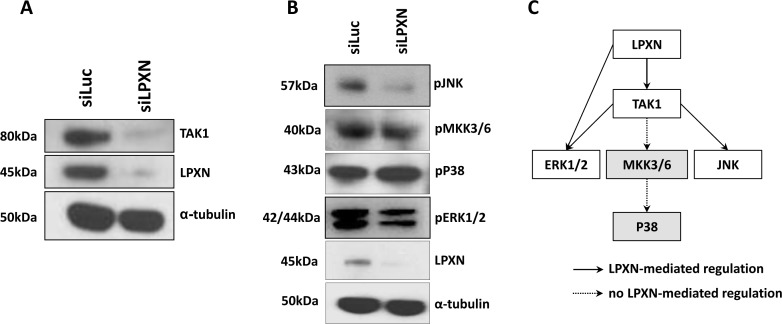
P38 is not involved in LPXN-mediated signaling P38 and ERK1/2 are each capable of regulating the actin affinity of l-CaD by phosphorylation. To determine which protein is mediating the phosphorylation of l-CaD in a LPXN-dependent manner, data from the Kinexus antibody array were analyzed. In this array, we found TAK1 to be downregulated after LPXN knockdown. (**A**) PC-3 cells were transfected with LPXN-specific siRNA and control siRNA (siLuc) and incubated for 48 hours before protein isolation. Western blotting using a specific antibody against TAK1 verified the results of the antibody array. (**B**) TAK1 can induce signaling via the MKK3/6-P38 pathway or via the JNK pathway. Therefore, we checked the phosphorylation status of these two proteins in PC-3 cells after the downregulation of LPXN expression. After serum-starved cells were stimulated with 10% FCS for 10 min, we could not observe a reduction in phosphorylation of MKK3/6 and P38. Instead, phosphorylation of ERK and JNK was reduced. Thus, we concluded that P38 is not the kinase involved in LPXN-mediated phosphorylation of l-CaD.

To investigate which kinase phosphorylates CaD in a LPXN-dependent manner, we first analyzed the signaling pathways influenced by LPXN using the antibody array KAM 850 (Kinexus) with protein lysates from PC-3 cells after LPXN knockdown together with protein from control transfected PC-3 cells. We found the TGF-beta activated kinase 1 (TAK-1), among others, to be downregulated in response to LPXN knockdown (Table 1, Figure 6A). TAK-1 has been shown to activate JNK and p38 MAPK through phosphorylation [21, 22]. Subsequent western blot analyses showed no changes in the phosphorylation status of p38 MAPK, whereas phosphorylation of JNK and ERK was reduced after LPXN knockdown (Figure 6B). This is confirmed by the fact that the phosphorylation status of the p38 MAPK upstream pathway protein MKK3/6 was also not altered after LPXN knockdown (Figure 6B and 6C).

**Table 1:** Overview of the results obtained from the antibody array KAM 850 (Kinexus) of PC-3 cells after LPXN knockdown in comparison to luciferase (control) transfected cells. The table only displays proteins that changed their expression levels. Interestingly, TAK1 was downregulated in response to LPXN knockdown, which is known to activate downstream signaling pathways such as the JNK or p38 MAPK pathway

Symbol	Protein (complete name)	*fold change* siLPXN/siLuc
KDEL receptor 1	ER lumen protein retaining receptor 1	2.39
cyclin D1	Cyclin D1 (PRAD1)	2.16
Hsp90	Heat shock 90 kDa protein alpha/beta	2.12
Hsp90	Heat shock 90 kDa protein alpha/beta	2.10
PKCb1	Protein-serine kinase C beta 1	2.04
p21 CDKI1	cyclin-dependent kinase inhibitor 1 (MDA6)	0.55
CDK1 (CDC2)	Cyclin-dependent protein-serine kinase 1	0.53
PKBb (Akt2)	Protein-serine kinase B beta	0.51
Yes	Yamaguchi sarcoma proto-oncogene-encoded tyrosine kinase	0.49
TAK1	TGF-beta-activated protein-serine kinase 1	0.47
GSK3a	Glycogen synthase-serine kinase 3 alpha	0.30

### LPXN interacts with extracellular signal-regulated kinase (ERK)

**Figure 7:**
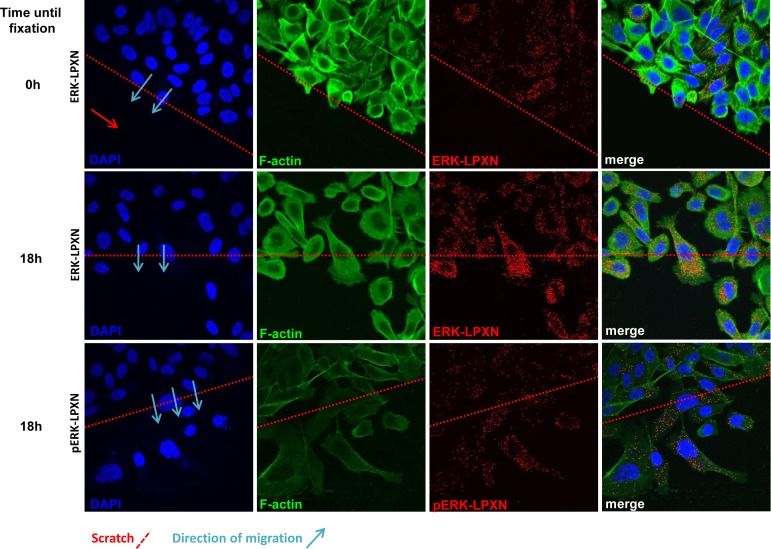
Interaction of ERK and LPXN during PCa cell migration ERK-LPXN interactions during migration are shown by a combination of scratch and proximity ligation assays on PC-3 cells. Cells were grown to confluence, and a scratch was applied to the cell layer. Cells were fixed immediately (0 hours) or after 18 hours, and proximity ligation assays were performed. Few interactions of ERK and LPXN were observed in confluent cells (upper row), whereas migrating cells showed increased interactions of ERK and LPXN (middle row) as well as phospho-ERK and LPXN (lower row). This interaction pattern was similar to that observed for the CaD-LPXN interaction during migration.

Because p38 MAPK was not affected by LPXN knockdown and ERK has previously been described to be associated with migration-dependent phosphorylation at the actin binding sites of CaD, we hypothesized that ERK is the kinase responsible for LPXN-mediated phosphorylation of CaD during cell migration. To test our hypothesis, we investigated a putative interaction between ERK and LPXN. Using *in situ* PLA, we were able to detect interaction of LPXN and ERK in PC-3 and DU 145 cells (Figure 7).

Having shown a clear interaction between LPXN and phospho-CaD during migration of PC-3 cells and an interaction between LPXN and ERK, we examined whether the LPXN-ERK interaction is also induced by migration. Scratches were applied to confluent PC-3 cell layers, and cells were fixed immediately or after 18 hours. PLAs were performed using specific antibodies against LPXN and ERK. Similar to the CaD-LPXN interaction, we observed an increase in the interaction of LPXN and ERK in migrating cells compared to confluent or non-migrating cells (Figure 7). Interestingly, we also found the same interaction pattern of LPXN with activated ERK (p-ERK) (Figure 7), supporting our hypothesis that LPXN mediates ERK-dependent phosphorylation of CaD.

### LPXN mediates the phosphorylation status of CaD through interaction with ERK

**Figure 8:**
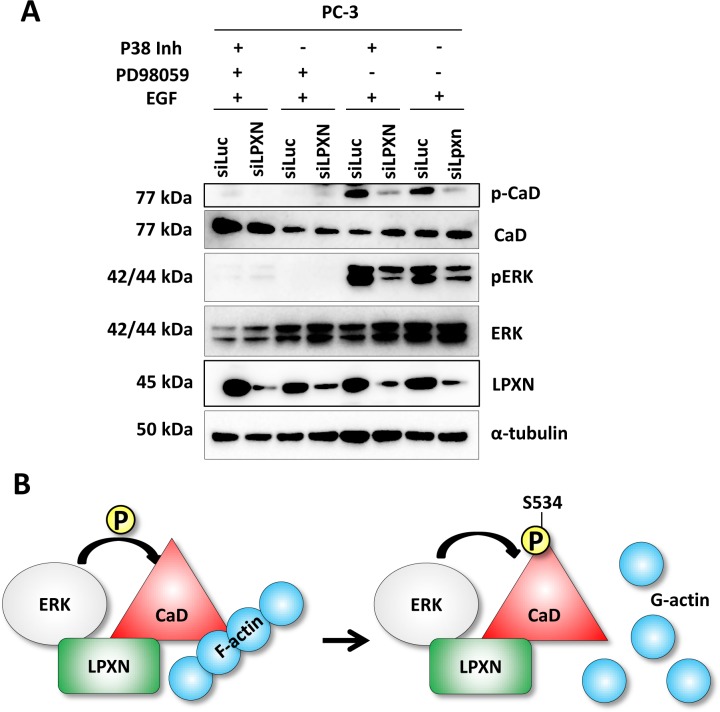
LPXN mediates CaD phosphorylation through interaction with ERK (**A**) PC-3 cells were transfected with LPXN-specific siRNA or as a control with luciferase-specific siRNA. After 48 hours, cells were serum-starved overnight and then treated with the p38 inhibitor SB203580, the MEK-1 inhibitor PD98059, or both for one hour before stimulation with EGF. Knockdown of LPXN reduced phosphorylation levels of CaD and notably of ERK. Inhibition of MEK-1 using PD98059 abolished the activation of ERK and the phosphorylation of CaD. Inhibition of p38 had no influence on the phosphorylation of CaD. With the p38 inhibitor, only LPXN knockdown diminished CaD phosphorylation. (**B**) Scheme of the hypothesized mechanism of LPXN-mediated phosphorylation of CaD through ERK. In response to migration-promoting signals, LPXN recruits ERK to phosphorylate CaD (at S534 for l-CaD; S789 for h-CaD). Once CaD is phosphorylated, it detaches from F-actin, leading to dynamic actin structures.

To further clarify whether ERK is the kinase that is recruited to phosphorylate CaD during migration, we treated PC-3 cells with the P38 inhibitor SB203580 or the MEK1 (mitogen-activated protein kinase kinase 1)-specific inhibitor PD98059 after LPXN knockdown. PD98059 blocks activation of ERK by preventing phosphorylation through its upstream kinase MEK1. Compared with the control transfected cells (siLuc), no reduction of CaD phosphorylation was observed after treatment with the P38 inhibitor. Only the knockdown of LPXN expression reduced CaD phosphorylation in P38 inhibitor-treated cells. Instead, CaD phosphorylation was markedly decreased as a consequence of ERK inhibition. Furthermore, LPXN knockdown decreased pERK levels but did not enhance the effect of PD98059 on CaD phosphorylation. (Figure 8A). This observation supports a model in which P38 is not important for CaD phosphorylation in PCa cells and LPXN regulates the phosphorylation of CaD via ERK; this phosphorylation affects the binding of CaD to actin and thereby regulates actin-cytoskeletal dynamics (Figure 8B) and the migratory ability of PCa cells.

## DISCUSSION

Cell adhesion is fundamentally important in development and tissue homeostasis. Deregulation of this complex process leads to detachment of tumor cells from the tissue of origin and is an important determinant of their metastatic capacity. In previous studies, we showed that LPXN directly influences the migration and invasion of PCa cells [7] and that overexpression of LPXN in the prostate increases progression and metastasis of TRAMP tumors [8]. In the present study, we report that LPXN has dramatic effects on the adhesion of PC-3 and DU 145 cells. Cells with LPXN knockdown showed reduced cell size and number on multiple extracellular matrix (ECM) substrates. The most dramatic effect was observed on fibronectin. After two hours, siLPXN-transfected PCa cells showed significantly reduced spreading, whereas after 24 hours, they were no longer distinguishable from control cells.

Taken together, these results suggest that LPXN might have important roles in the formation of focal adhesion sites. This is supported by the fact that LPXN was originally found to be expressed in highly motile cells such as macrophages, osteoclasts and leukocytes, whereas in other tissues, LPXN was not detectable [4, 5]. In addition, LPXN is overexpressed in several cancer cell lines [23]. Thus, an enhancement of adhesion by LPXN is not likely in these cells as was hypothesized for MDA-MB-231 breast cancer cells by Chen et al. [23]. All of these highly motile cells require a high focal adhesion turnover for rapid migration. From the aforementioned studies, there seems to be a correlation between LPXN expression and cell motility. This fact is also supported by previous studies from our group [7]: we could demonstrate a correlation of LPXN expression with the Gleason score of human prostate carcinomas. We therefore suggest that LPXN is involved in the process of cell adhesion and could be relevant for the formation of focal adhesions. However, because of the conflicting data and to further confirm our hypothesis of LPXN function in the formation of focal adhesions, additional studies must be conducted. These studies might also address whether LPXN is required for later focal adhesion maintenance.

To leave their place of origin, tumor cells must lose adhesion as well as migrate and even penetrate and invade surrounding tissues. Recently, we demonstrated elevated expression levels of LPXN in approximately 20% of prostate cancer patients and showed that knockdown of LPXN by RNAi reduces migration and invasion of PC-3 and DU 145 PCa cell lines [7]. However, the molecular mechanism behind this LPXN-mediated migration and invasion remained elusive. LPXN has already been described to interact with several proteins such as sarcoma (SRC) [9], protein tyrosine kinase 2 (PYK2) [4], and protein tyrosine phosphatase (PTP)-PEST [24]. However, none of these interactions have been directly linked to migration and invasion in PCa cells. A very early and essential feature of migrating cells is the remodeling of the actin cytoskeleton. Therefore, we were interested in putative interaction partners that could link LPXN to cytoskeletal rearrangements.

Here, we identified the actin-binding protein caldesmon (CaD) as an interaction partner of LPXN. In addition, these proteins showed similar expression patterns and co-localization on the subcellular level was observed in PC-3 and DU 145 cells. CaD is known to play an essential role in actin cytoskeletal dynamics and cell contraction, but little is known regarding its functions in cancer cells. Most evidence today is based on studies that have been conducted on h-CaD, which is the high molecular mass isoform of l-CaD and is exclusively expressed in smooth muscle cells, where it takes part in muscle contraction. Because both isoforms are generated from the same gene, their domain sequences are identical, and their biochemical characteristics are expected to be quite similar. h-CaD has been reported to have tumor-supporting [25] as well as tumor-suppressing [26] functions and is also used as a diagnostic marker for smooth muscle tumors [27].

In our experiments, we show increased migration and invasion of PC-3 and DU 145 cells after CaD knockdown. This data is supported by two recent independent studies that achieved the same results in MDA-MB-231 breast cancer cells [28] and AGS and FU97 gastric cancer cells [29]. Interestingly, the latter study further showed that CaD expression is decreased in several gastric cancer cell lines. Additionally, our findings are clearly supported by several investigations that revealed CaD as an actin-binding and stabilizing protein [30-33]. Moreover, CaD is known to act on multiple actin-associated proteins such as fascin [34], filamin [35] and cofilin [36], and it can also inhibit actin polymerization by acting on the Arp2/3 complex [10]. This inhibition of the Arp2/3 complex also negatively influences podosome formation, which is important for cell invasion and highlights the function of CaD as a podosome inhibitor [26, 37]. These highly dynamic structures exhibit functions in cell adhesion and in matrix metalloprotease (MMP) secretion, which is essential for focal extracellular matrix (ECM) degradation and is key to invasion of the surrounding tissue.

Taken together, CaD represents a suitable candidate protein that controls actin stabilization and thereby migration and invasion dynamics downstream of LPXN. In our model, LPXN represents a mediator and regulator of CaD phosphorylation through interaction with ERK, a mechanism that may explain the aggressive growth of LPXN-overexpressing PCa cells.

Several lines of evidence indicate that the function of CaD is regulated by calmodulin (CaM) binding and phosphorylation [18, 38]. A number of kinases have been described that phosphorylate h-CaD at various residues [39-43]. The major phosphorylation sites in h- and l-CaD are ERK-specific sites and are phosphorylated in response to pro-migration stimuli [44]. The modulatory role of ERK-dependent phosphorylation of CaD in cell motility and migration has already been reported [44]. These phosphorylation sites reside in the C-terminal region that also contains the actin binding domains [18, 45]. Consequently, ERK-mediated phosphorylation of l-CaD at S534 (corresponding to S789 in h-CaD) results in reduced actin binding and in actin stress fiber disassembly, which will finally enable migration [17].

Interestingly, the p38 MAPK has also been reported to phosphorylate CaD at these specific sites. However, we show that p38 MAPK activity was not affected by downregulation of LPXN, whereas the phosphorylation levels of ERK were reduced after LPXN knockdown in PC-3 and DU 145 cells. This is consistent with a study that showed no effect of the p38 MAPK-specific inhibitor, SB203580, on phosphorylation of S789 in h-CaD (corresponding to S534 in l-CaD) in rat vascular smooth muscle cells [19].

According to these data and our findings, p38 MAPK might have distinct functions in CaD phosphorylation but does not play a role in the migration-dependent phosphorylation of CaD. Therefore, we excluded p38 MAPK from our further analyses and focused on ERK-mediated phosphorylation of CaD. In the present study, we hypothesized a possible mechanism for LPXN-mediated migration of PCa cells, in which LPXN acts as an adapter protein that mediates phosphorylation of l-CaD at S534 by the recruitment of ERK (Figure 7B). In the PCa cell line PC-3, we demonstrated reduced phosphorylation of S534 in l-CaD after LPXN knockdown. Using a MEK-1-specific inhibitor, PD98059, we reduced pCaD levels even in the presence of LPXN, demonstrating that CaD phosphorylation at S534 is ERK-specific in PCa cells.

In summary, LPXN plays an important role in the adhesion of PCa cells. We found that LPXN acts as a mediator of ERK-dependent CaD phosphorylation and might thereby regulate actin-cytoskeletal dynamics. Pathological conditions caused by LPXN overexpression might thus promote migration and invasion of PCa cells.

## MATERIALS AND METHODS

### Cell culture, treatment and transient transfection

LNCaP, PC-3 and DU 145 cells (ATCC, Rockville, USA) were grown in RPMI 1640 medium (PAN-System, Nuremberg, Germany) containing 10% fetal calf serum and 1.2% penicillin/streptomycin. Transient transfection was carried out using the X-tremeGENE HP DNA Transfection Reagent (Roche, Mannheim, Germany) according to the manufacturer's instructions. For inhibition of p38 and MEK1/2, cells were serum-starved overnight and treated with 10 μM SB203580 or 20 μM PD98059, respectively. For stimulation of CaD phosphorylation, cells were stimulated with 10% FCS in the medium for 10 min, and protein was subsequently isolated.

### Cell adhesion assay

Before cells were plated, glass slides were coated with 20 μg/cm² bovine serum albumin, 1% gelatin, or rat-tail collagen for 1 hour or with fibronectin (FN) (Roche, Mannheim, Germany) according to the manufacturer's instructions. PC-3 and DU 145 cells were plated on coated and uncoated glass slides (BD Pharmingen™, Heidelberg, Germany). Adhesive cells were washed once with DPBS (PAN) and fixed after 1, 2 or 24 hours, as indicated. After staining of the actin cytoskeleton using fluorescein isothiocyanate (FITC)-conjugated phalloidin (Sigma-Aldrich, St. Louis, MO), cells were counted and cell size was determined by fluorescent microscopy (Olympus BX60) with CellSense® software.

### Generation of plasmid constructs

The full-length cDNA of human Leupaxin (NM_004811) was cloned in frame into the *EcoR*I and *Xho*I restriction sites of the pCMV-Myc vector (Clontech, Heidelberg, Germany) to produce a Leupaxin-Myc fusion protein. Likewise, full-length cDNA of human l-Caldesmon (NM_004342) was cloned into the pEGFP-C1 vector using the *Xho*I and *EcoR*I restriction sites and into pCMV-Myc vector using the *Sfi*I and *Xho*I restriction sites (both Clontech, Heidelberg, Germany) to produce EGFP-Caldesmon and Caldesmon-Myc fusion proteins, respectively. Fusion constructs were expressed in PC-3, DU 145 and LNCaP cells to analyze subcellular localization. For GST pulldown, full-length cDNA of human Leupaxin as well as LD motifs (aa 32-176) and LIM (aa 173-417) domains were cloned into the *EcoR*I and *Xho*I sites of the bacterial expression vector pGEX-4T3 (GE Healthcare, Munich, Germany) to produce GST-LPXN, GST-LPXN-LD and GST-LPXN-LIM fusion proteins.

### Yeast two-hybrid experiments

Yeast two-hybrid screening was performed using LPXN as bait (pGBKT7-LPXN) and the prostate cDNA library cloned into pGADT7 (Clontech, Heidelberg, Germany). Yeast colonies were selected on minimal synthetic dropout (SD) plates lacking amino acids leucine, tryptophan, histidine and adenine (SD –LTHA). Growing yeast colonies were used to isolate plasmid DNA, which was sequenced using vector specific primers. The direct yeast two-hybrid experiments were carried out as described previously [7]. The complete open reading frames of human leupaxin (NM_004811) and human caldesmon (NM_004342) were cloned into the pGBKT7 vector and the pGADT7 vector (both Clontech, Heidelberg, Germany), respectively. To assay the protein-protein interactions, co-transformation of the constructs into the yeast host strain AH109 was performed. Co-transformants were selected on SD –LTHA medium containing 80 mg/l X-Gal (ICN).

### Co-immunoprecipitation

For co-immunoprecipitation, a pCMV-Myc-CaD or pCMV-Myc-LPXN construct, which contained either the full-length cDNA of CaD (NM_004342) or LPXN (NM_004811), respectively, was generated and transiently transfected into PC-3 cells. For immunoprecipitation, cell lysate of PC-3 cells was incubated with an anti-CaD or anti-LPXN antibody followed by a western blot analysis using a LPXN-specific or CaD-specific antibody, respectively.

### GST-pulldown

GST-fusion proteins were expressed in *E. coli* DH5α and then purified. Concurrently PC-3 cells were transfected with pEGFP-Caldesmon for 48 h to achieve overexpression of CaD. Isolated protein and the purified GST-fusion proteins were incubated together. The interaction of leupaxin and CaD permits binding of the (GST-LPXN)-(EGFP-CaD) complex to the glutathione-Sepharose. As a negative control, protein from transfected PC-3 cells was incubated with GST protein. After elution of the bound protein, the precipitates were immunoblotted using a CaD-specific antibody. The success of the purification was checked using an anti-GST antibody.

### Northern blot

Total RNA was isolated from PC-3, DU 145 and LNCaP cells with the RNeasy Mini Kit (Qiagen, Hilden, Germany) according to the manufacturer's instructions and used for subsequent northern blot analysis, as previously described [46]. The (^32^P)-labeled cDNA probes for human *β*-*actin* and human *CALD1* (ImaGene, Berlin, Germany) were hybridized to the membrane in Rapid-hyb buffer (GE Healthcare, Munich, Germany) at 65°C for 16 h. The filters were washed, and the hybridization signals were quantified with a Molecular Imager FX using Quantity One software (Bio-Rad, Munich, Germany).

### Western blot analyses

Western Blot analyses were carried out as previously described [7]. Primary antibodies used were as follows: anti-purified l-CaD (BD Pharmingen™, Heidelberg, Germany), anti-leupaxin 283 G (kindly provided by Eli Lilly & Co, Indianapolis, Indiana, USA), anti-α-tubulin (clone B-5-1-2) (Sigma–Aldrich, St. Louis, MO), anti-Tak-1 #4505, anti-phospho-MKK3/6 (Ser189/207) 22A8, anti-phospho-JNK (Thr183/Tyr185) #9251 (all from Cell Signaling, Danvers, MA, USA), anti-phospho-Caldesmon (Ser789), anti-phospho-p38 (Tyr-182)-R sc:7975-R (both from Santa Cruz Biotechnology, Heidelberg, Germany). The following secondary antibodies were used: peroxidase-conjugated AffiniPure rabbit anti-mouse IgG and goat anti-rabbit IgG (Dianova, Hamburg, Germany). The signals were captured using a FluorChem Q imaging system (Biozym Scientific GmbH, Hessisch Oldendorf, Germany) and analyzed using the FluorChem Q SA Software (Biozym Scientific GmbH).

### Immunocytochemistry

Prostate cancer cells were cultured for 24 hours at 37°C and 5% CO_2_ on cell culture slides. The cells were then fixed and treated as described previously [8]. The following specific antibodies were used: mouse monoclonal purified anti-l-Caldesmon (BD Pharmingen™, Heidelberg, Germany), rabbit polyclonal anti-Leupaxin, anti-cmyc and sheep anti-mouse IgG-Cy3 (all Sigma–Aldrich, St. Louis, MO). F-actin was stained using fluorescein isothiocyanate (FITC)-conjugated phalloidin (Sigma–Aldrich, St. Louis, MO). All images were captured on a FluoView1000 confocal laser-scanning microscope (Olympus, Hamburg, Germany).

### Proximity ligation assay

*In situ* proximity ligation assays (PLAs) were performed using the DuoLink® II Fluorescence Kit (Olink® Bioscience, Uppsala, Sweden) according to the manufacturer's instructions. Briefly, fixed cells were blocked with 3% BSA in PBS for 30 min at 37°C. All subsequent incubation steps were performed in a humidity chamber. Cells were incubated with specific primary antibodies raised in different species at suitable concentrations to detect proteins of interest. Secondary antibodies conjugated to specific plus and minus oligonucleotide probes, as indicated, were incubated with the reaction for 1 hour at 37°C. The linker oligonucleotide sequence and ligase were added and incubated for 30 min at 37°C to prepare for rolling-circle amplification of the annealed PLA probes. Rolling-circle amplification was carried out for 100 min at 37°C. Finally, the amplified sequences were detected using fluorescently labeled oligonucleotides. Protein proximity is indicated by distinct fluorescent spots analyzed by fluorescent microscopy using the FluoView1000 confocal laser-scanning microscope (Olympus, Hamburg, Germany). Antibodies to detect Leupaxin-Caldesmon/pS789-Caldesmon and Leupaxin-ERK/pERK interactions were purchased as follows: polyclonal anti-Leupaxin N-terminal SAB4200010 (Sigma-Aldrich, St. Louis, MO); monoclonal mouse anti-purified l-Caldesmon (BD Pharmingen™, Heidelberg, Germany), polyclonal rabbit anti-*p*-Caldesmon (Ser789) (Santa Cruz Biotechnology, Heidelberg, Germany); rabbit polyclonal p44/42 MAPK (ERK1/2) and rabbit monoclonal phospho-p44/42 MAPK (ERK1/2) (Thr202/Tyr204) D13.14.E4 (both from Cell Signaling).

### RNA interference

Transfection of PC-3 and DU 145 cells was accomplished as previously described [8]. Additional siRNA duplexes with the following target sequences were used: Cald1-A 5′-ACAUAUUGAUACCUAUCUGCCAUGU-3′; Cald1-B 5′-CAGAAGGAGUUCGACCCAACAAUAA-3′ (stealth siRNA, Invitrogen). Control cells were transfected with siRNA duplex oligonucleotides against the firefly luciferase gene. After 72 hours, cells were collected and used in the subsequent experiments.

### Migration and matrigel invasion assays

*In vitro* cell migration was analyzed as described previously [8]. In brief, siRNA-transfected PCa cells were plated on Millicell cell culture inserts (Millipore, Massachusetts, USA). After 24 hours, cells were stained using the Diff-Quik staining kit (Medion Diagnostics, Switzerland) and photographed under an inverted microscope (Olympus IX81; Olympus, Hamburg, Germany). *In vitro* cell invasion of PC-3 and DU 145 cells was determined in BioCoat Matrigel Invasion Chambers (BD Pharmingen™, Heidelberg, Germany) as described previously [8].

### Proliferation assay

For these assays, 3 × 10^3^ siRNA-transfected PC-3 or DU 145 cells were plated in 96-well plates, and proliferation was determined using the MTS assay (CellTiter 96® AQueous Non-Radioactive Cell Proliferation Assay (MTS) Promega) according to the manufacturer's instructions.

## SUPPLEMENTARY MATERIAL AND FIGURES


