# Supplementary material for: Leupaxin stimulates adhesion and migration of prostate cancer cells through modulation of the phosphorylation status of the actin-binding protein caldesmon
Source: Oncotarget. 2015 Apr 12;6(15):13591–606. doi: 10.18632/oncotarget.3792 (PMC4537036; doi:10.18632/oncotarget.3792)
Supplement: Supplementary file 1 [file oncotarget-06-13591-s001.pdf]

Leupaxin stimulates adhesion and migration of prostate cancer cells through modulation of the phosphorylation status of the actin-binding protein caldesmon

Supplementary Material

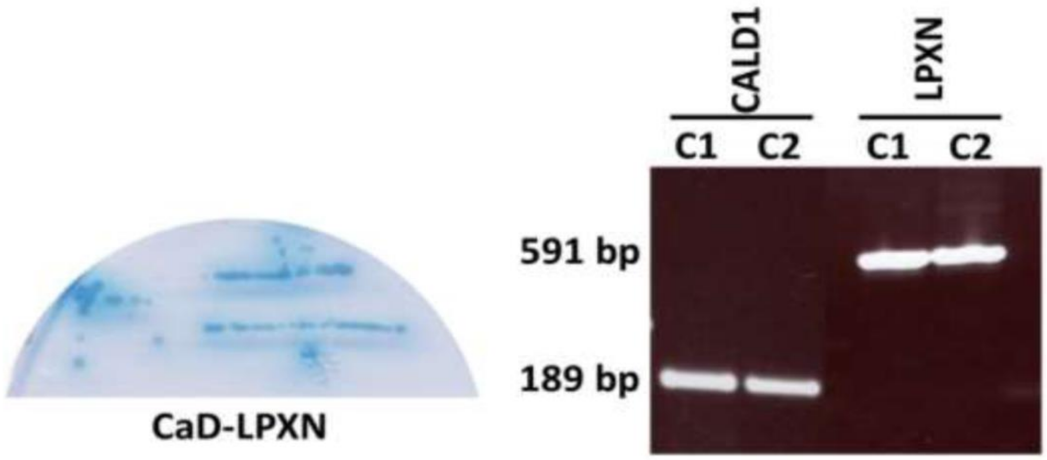

Supplement Figure 1: Interaction of CaD and LPXN in a direct yeast two-hybrid experiment. The direct interaction of LPXN with CaD was verified using a yeast two-hybrid experiment. Yeast cells were transformed with full length plasmids pGBKT7-CALD1 and pGADT7-LPXN and plated on dropout plates with α-Gal added. Subsequently, PCR analyses on two different yeast clones confirmed the presence of the plasmids pGBKT7-CALD1 (189 bp) and pGADT7-LPXN (591 bp) and consequently the interaction of LPXN and CaD.

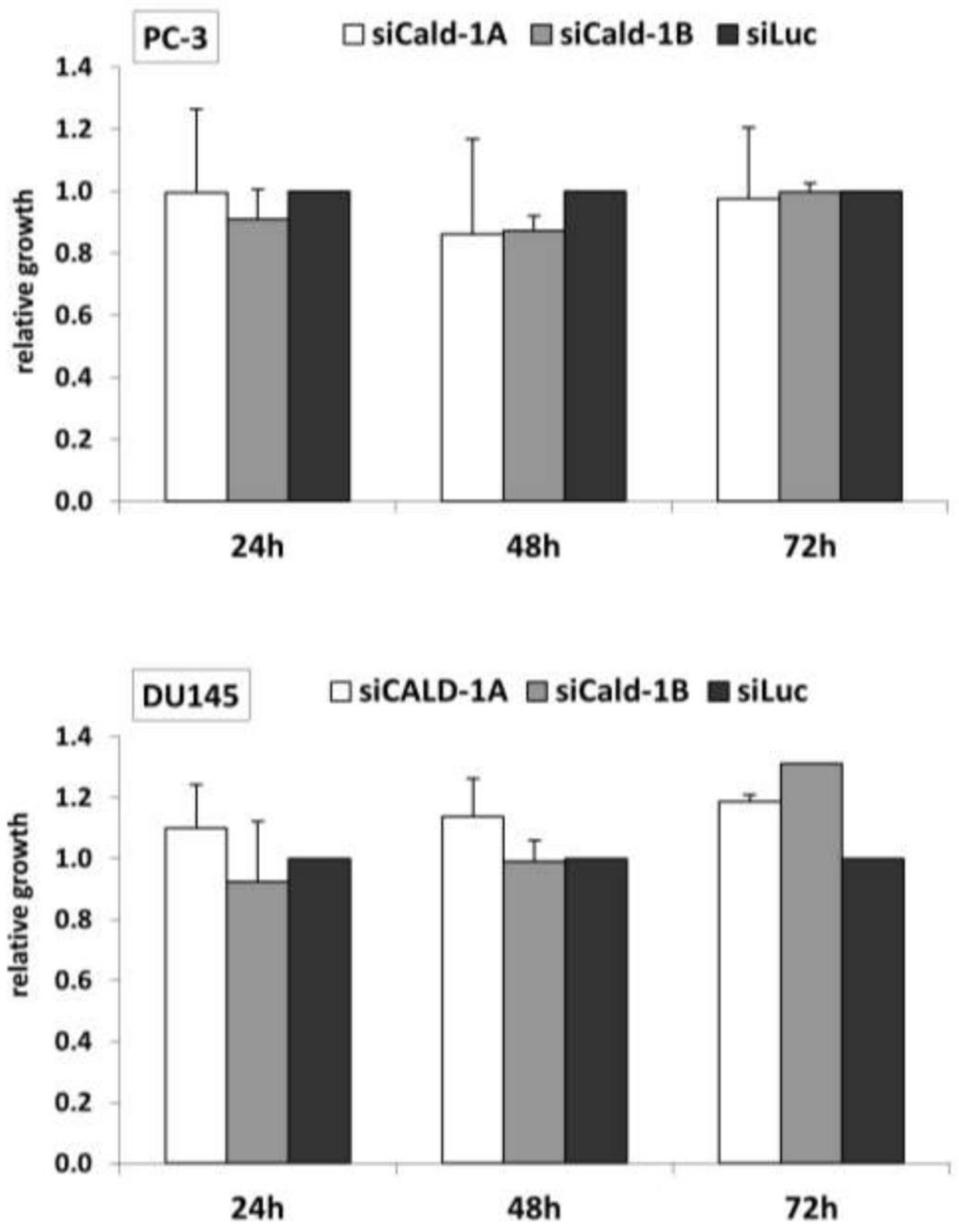

Supplement Figure 2: Proliferation of PCa cells PC-3 and DU 145 after knockdown of l-CaD expression. PCa cell lines PC-3 and DU 145 were transfected with CALD1-specific siRNAs siCald-1A and siCald-1B and as control with luciferase siRNA siLuc. 24h after transfection cells were plated for MTS-assay in triplicate. Depicted time points relate to the start of proliferation measurement. Data are the mean ±s.d. from three independent experiments.

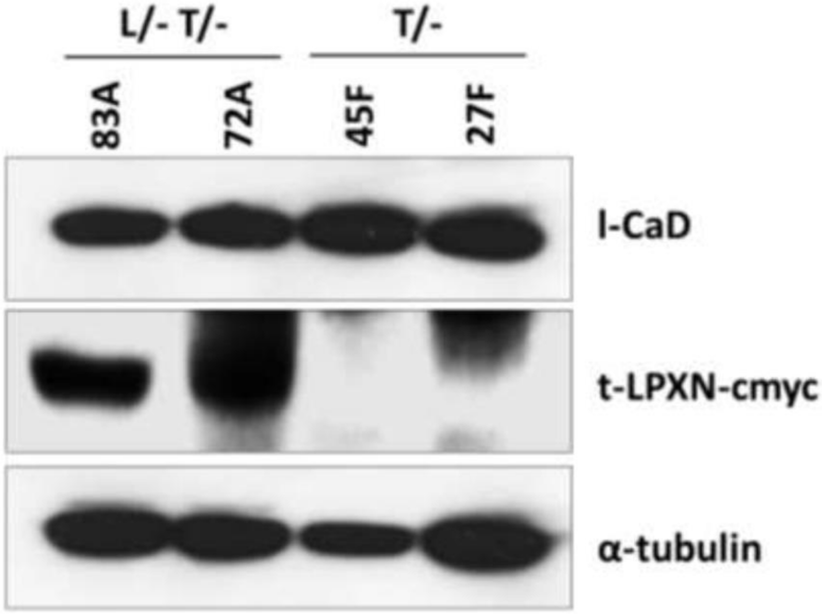

Supplement Figure 3: Expression of l-Cad in primary prostate cancer cell lines of the transgenic mouse model LPXN/TRAMP. To analyze the impact of LPXN on l-CaD protein isolated from primary prostate tumor cells of LPXN/TRAMP double transgenic (83A and 72A) and single transgenic TRAMP mice (45F and 27F) was immunoblotted using a l-CaD-specific antibody. l-CaD is equally expressed.
